# The tale of microencapsulated rifampicin: is it useful for the treatment of periprosthetic joint infection?

**DOI:** 10.1007/s00264-021-05290-0

**Published:** 2022-01-06

**Authors:** Irene Isabel López-Torres, Javier Vaquero-Martín, Ana-Isabel Torres-Suárez, Federico Navarro-García, Ana-Isabel Fraguas-Sánchez, Víctor Estuardo León-Román, Pablo Sanz-Ruíz

**Affiliations:** 1 Traumatology and Orthopaedic Surgery department, Fundación Jiménez Díaz Hospital, Av. Reyes Católicos 2, 28040 Madrid, Spain; 2 Traumatology and Orthopaedic Surgery department, Gregorio Marañón General Hospital, C/Doctor Esquerdo 46, 28007 Madrid, Spain; 3 Surgery Department, Faculty of Medicine, Complutense University of Madrid, Plaza Ramón Y Cajal S/N, 28040 Madrid, Spain; 4 Galenic Department, Faculty of Pharmacy, Complutense University of Madrid, Plaza Ramón Y Cajal S/N, 28040 Madrid, Spain; 5 Microbiology and Parasitology Department, Faculty of Pharmacy, Complutense University of Madrid, Plaza Ramón Y Cajal S/N, 28040 Madrid, Spain; 6 Traumatology and Orthopaedic Surgery department, Villalba General Hospital, Carretera de Alpedrete a Moralzarzal, M-608 km 41, Collado Villalba, Madrid, Spain

**Keywords:** Prosthetic joint infection, Rifampicin, Bone cement, Rabbit

## Abstract

**Purpose:**

Microencapsulation techniques have allowed the addition of rifampicin to bone cement, but its in vivo efficacy has not been proven. The aim of our study is to determine the superiority of cement containing gentamicin and rifampicin microcapsules in the treatment of PJI versus cement exclusively containing gentamicin.

**Methods:**

An *S. aureus* PJI was induced in 15 NZW rabbits. A week after inoculation, the first stage of replacement was carried out, and the animals were divided into two groups: group R received a spacer containing gentamicin and rifampicin microcapsules, and group C received a spacer containing gentamicin. Intra-articular release curve of rifampicin and infection and toxicity markers were monitored for four weeks post-operatively, when microbiological analysis was performed.

**Results:**

The microbiological cultures showed a significantly lower growth of *S. aureus* in soft tissue (2.3·10^4^ vs 0; *p* = 0.01) and bone (5.7·10^2^ vs 0; *p* = 0.03) in the group with rifampicin microcapsules. No differences were found in systemic toxicity markers. Rifampicin release from the cement spacer showed higher concentrations than the staphylococcal MIC throughout the analysis.

**Conclusion:**

The in vivo analyses demonstrated the superiority of cement containing gentamicin and rifampicin microcapsules versus the isolated use of gentamicin in the treatment of PJI in the rabbit model without serious side effects due to the systemic absorption of rifampicin. Given the increasing incidence of staphylococci-related PJI, the development of new strategies for intra-articular administration of rifampicin for its treatment has a high clinical impact.

## Introduction

Periprosthetic joint infection (PJI) is a challenging complication with rising incidence due to the increased life expectancy and the functional demand of young patients. Currently, the incidence of PJI is 2–2.4%, but it is estimated to increase and become the main cause of revision surgery [1–3]. The main causative agents are coagulase-negative staphylococci and *Staphylococcus aureus*, accounting for 39 and 31% of cases, respectively. When they encounter with prosthetic material, they adhere to it forming a biofilm, increasing the appearance of resistances to antibiotics by acting as a physical barrier to its penetration and inhibiting the immune response of the host. Thus, the presence of a biofilm causes the effective doses of antibiotic to multiply by 200 to 1000 times [4–7].

Currently, two-stage replacement is considered the gold standard for the treatment of chronic PJI and involves the removal of the prosthesis and temporary placement of a cement spacer containing antibiotics [8]. This technique allows high intra-articular antibiotic concentrations, minimising toxic systemic side effects [9, 10]. The surgery is followed by a prolonged systemic antibiotic therapy, being rifampicin in combination with quinolones, the preferred treatment for PJI caused by biofilm-forming microorganisms [11–13]. Rifampicin has been shown to be effective against all forms of staphylococci present in PJI pathogenesis, that is, intracellular, planktonic, and sessile forms, so its addition to bone cement would be invaluable for the treatment of these infections. The main problem is that its addition to bone cement alters the polymerisation process, giving rise to an incompletely set cement not suitable for use in clinical practice [14, 15]. Therefore, nowadays the only route of administration is systemic, leading to significant side effects derived from the high doses necessary to achieve effective levels of antibiotic in the joint.

In 2016, our research group designed a controlled release system for rifampicin in the form of alginate microcapsules, which showed to preserve the mechanical properties of bone cement with an optimum rifampicin release profile during in vitro studies [16–18]. The incorporation of these rifampicin microcapsules into the cement spacer would allow the prolonged release of antibiotic, increasing its efficacy and reducing the side effects of its systemic distribution. Therefore, the aims of the present in vivo testing are to determine the effectiveness of bone cement containing rifampicin microcapsules, to establish the in vivo safety profile of microencapsulated rifampicin and to register the rifampicin intra-articular release profile.

## Materials and methods

### Animals and surgical procedures

**Fig. 1:**
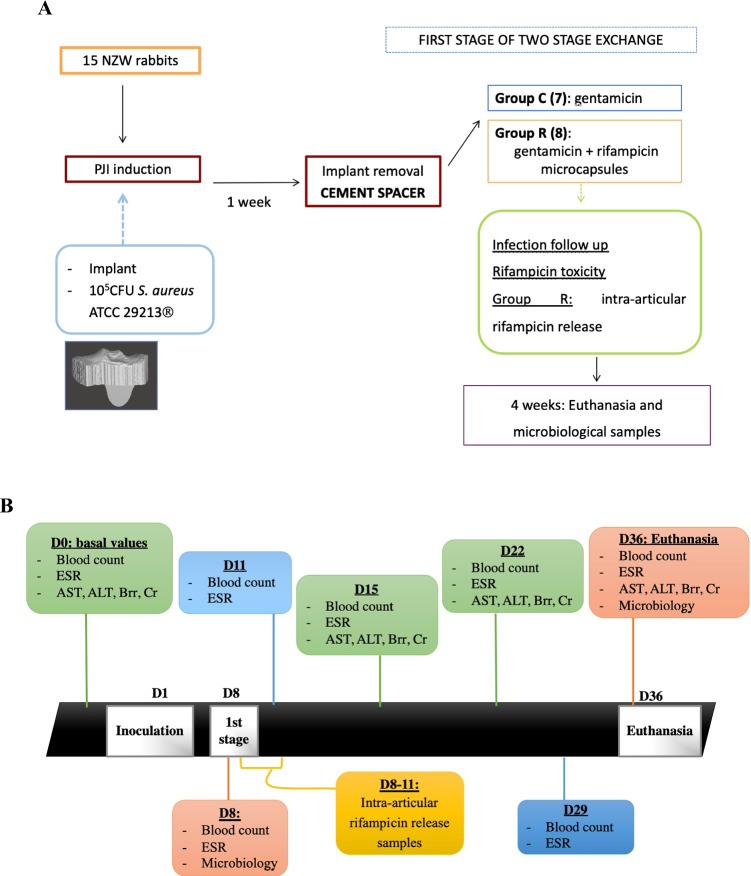
**A** Study protocol diagram. **B** Graphic representation of the timeline of blood and intra-articular and microbiological samples taken throughout the study

To carry out the trial, 15 female New Zealand White rabbits (Granja San Bernardo, Spain) weighing about 3 kg each were used. Food and water were available ad libitum. The anaesthesia and analgesia protocol implied induction with a single dose of ketamine (50 mg/kg) and xylazine (10 mg/kg) and analgesia with subcutaneous meloxicam (1 mg/kg/24 h) for the first four postoperative days. All animals had their proximal tibial metaphysis replaced by a stainless steel insert designed by 3D printing according to the technique described in another study [19]. After implantation and closure of the arthrotomy, 1 mL of 10^5^ CFU from the methicillin-sensitive *Staphylococcus aureus* strain ATCC® 29,213™ was inoculated by intra-articular injection [20]. One week after the inoculation, the animals were re-anaesthetised to carry out the first stage of prosthetic replacement, intra-operative samples were taken to verify the presence of infection, the stainless steel insert was removed, and a cement spacer was fitted. The animals were then divided into two groups: group C (7 rabbits), in which the spacer contained gentamicin of routine clinical use (Palacos® R + G, Heraeus, Hanau, Germany), and group R (8 rabbits), in which a cement spacer containing gentamicin and 12.5% of rifampicin microcapsules was implanted. Rifampicin microcapsules [21] were sterilised by gamma radiation (25KGy) and analysed in terms of drug content (2.48 ± 1.05%) prior to use. The trial protocol is summarised in Fig. 1A.

### Analytical determinations

The defining parameters of infection and systemic toxicity of the rifampicin were monitored by analytical, weight, and temperature determinations before the start of the study, during the first stage of replacement, four days after replacement, and, subsequently, on a weekly basis until the fourth week after surgery (Fig. 1B). The diagnostic criteria for infection are summarised in Table 1 [20]. The evolution of the inflammatory parameters was defined according to variations in ESR, leukocyte count, platelet count, and percentage of serum lymphocytes. Four weeks after spacer implantation, the animals were sacrificed with an overdose of sodium pentobarbital, and samples of soft tissue (synovial and capsule) and bone were taken. The spacers were also removed, sonicated, and washed three times with sterile saline solution to eliminate planktonic bacteria. All these samples were treated using a previously described procedure [20] in order to obtain final microbiological culture samples.

**Table 1:** Diagnostic criteria of infection in the rabbit

At least one major criterion: -The presence of a fistula in contact with the articulation -At least two positive intra-articular cultures for *Staphylococcus aureus*
At least three minor criteria: -A leukocyte count above 9.7·10^3^/μL -Lymphopenia ≤ 30% -Thrombocytosis > 650·10^3^/μL -ESR > 5 mm -A positive culture

The systemic toxicity of the rifampicin was defined according to the development of the two major side effects that lead to the suspension of the administration in the clinical practice: hepatotoxicity and flu-like syndrome. Thus, the systemic toxicity of the rifampicin was determined based on the increased liver enzymes (AST, ALT), bilirubin, and renal function (creatinine) markers.

To define the intra-articular release curve for the rifampicin contained in the microcapsules, intra-articular samples were taken from group R animals at six, 24 hours, and daily after replacement surgery. This quantification was carried out by high performance liquid chromatography (HPLC) using a previously validated method.

### Statistical analysis

Sample size determination was carried out with Statgraphics Centurion XV® software (Statgraphics Technologies Inc., USA). Thus, for a level of significance of 0.05 and with a power of 90%, it was determined that it was necessary a minimum of 6 animals per group to carry out the study. Thus, 15 animals started the trial, considering that some animals would have to be discarded during the course of the trial. The statistical analysis of data was carried out with SPSS Statistics® software, version 22.0 for Mac (IBM, NY, USA), with *p*-values less than 0.05 being considered statistically significant. Comparison of qualitative variables was performed with the *χ*^2^ test and comparative before-after studies with the paired-samples *t*-test. Comparison of normal quantitative variables with dichotomous qualitative variables was carried out with Student’s *t*-test, while comparison of non-normal quantitative variables with dichotomous qualitative variables was carried out with the Mann–Whitney *U* test.

The study was developed after receiving approval from the Regional Ethical Committee (code: 10/143903.9/17).

## Results

All the animals recovered from the surgical procedures without incident. The efficacy of the intra-articular inoculation of *S. aureus* was 93.3%, with a PJI developing in 14 of the 15 animals, with these 14 being used to continue the study. Baseline determinations are resumed in Table 2.

**Table 2:** Baseline infectious and toxicity data and *p* values for each experimentation group

Basal measurement	$\overline{X }$(SD)		*p*
	Control	Rifampicin	
Haemoglobin (g/dL)	12.3 (0.29)	12.65 (0.51)	0.1
Leukocytes (·10^3^/mm^3^)	7.2 (1.2)	6.7 (0.67)	0.3
Lymphocytes (·10^3^/mm^3^)	4.7 (1.1)	4.6 (0.72)	0.7
Platelets (·10^3^/mm^3^)	267.2 (81.11)	294.37 (64)	0.4
ESR (mm)	2.5 (0.7)	3.4 (1.5)	0.4
AST (UI/L)	24.6 (20.99)	14.5 (4)	0.3
ALT (UI/L)	46.2 (16.6)	51.8 (14.8)	0.5
Bilirubin (mg/dL)	0.11 (0.04)	0.11 (0.04)	0.9
Creatinine (mg/dL)	0.84 (0.07)	0.84 (0.08)	0.9

### In vivo* microbiological effectiveness*

Four weeks after spacer implantation, the microbiological cultures of the samples taken from sacrificed animals showed a significantly lower growth of *S. aureus* in the group treated with cement containing rifampicin microcapsules (R) for the samples obtained from soft tissues and bone, with *p* = 0.01 and 0.03, respectively. No statistically significant differences were found in the microbiological count after sonication of the spacer (Table 3).

**Table 3:** Microbiological results (UFC) at the end of the study (4 weeks after the first stage of exchange)

Sample	$\overline{X }$(SD)		*p*
	Control	Rifampicin	
Spacer	1.09·10^2^ (SD 2.19·10^2^) UFC	3.6·10^2^ (SD 7.4·10^2^) UFC	0.71
Bone	5.7·10^2^ (SD 1.2·10^3^) UFC	0 UFC	0.03
Soft tissue	2.3·10^4^ (SD 5.6·10^4^) UFC	0 UFC	0.01

**Fig. 2:**
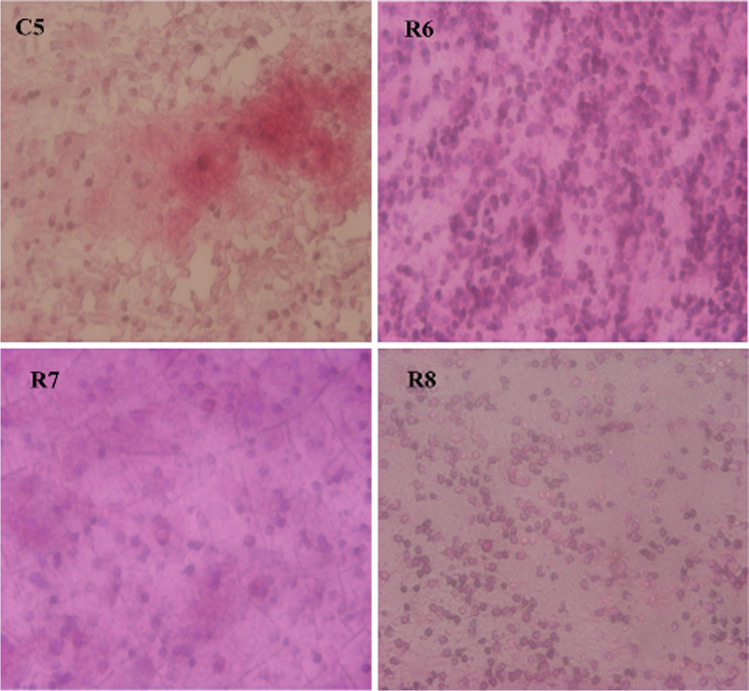
Histological analysis of synovial fluid in samples from the control (C) and rifampicin (R) groups. Haematoxylin eosin staining did not show differences between the inflammatory cells in both groups neither *S. aureus* grow in the R group

The synovial fluid histological analysis conducted in samples from control and rifampicin groups did not show differences between the inflammatory cells in both groups and did not show *S. aureus* growing with haematoxylin eosin straining (Fig. 2).

No statistically significant differences were found either between study groups in terms of weight, temperature, ESR, leukocyte count, platelet count, and percentage of lymphocytes in the blood at any time of measurement. Leukocyte count and percentage of lymphocytes in the blood showed statistically significant normalisation at the end of the study with respect to the postinoculation *S. aureus* values (Table 4).

**Table 4:** Evolution of infectious parameters

Parameter	$\overline{X }$(range)		*p*
	1 week postinoculation	4 weeks postinoculation	
Leukocytes (·10^3^/μL)	11.39 (8.5–19.3)	8.79 (5.9–12.6)	0.001
Lymphocytes (%)	32.14 (21.2–44.9)	44.92 (25.7–63.4)	< 0.001
Platelets (·10^3^/μL)	694 (412–882)	550.5 (314–1281)	0.2
ESR (mm)	5 (2–16)	2.2 (2–3)	0.21

### In vivo* safety profile of microencapsulated rifampicin*

**Fig. 3:**
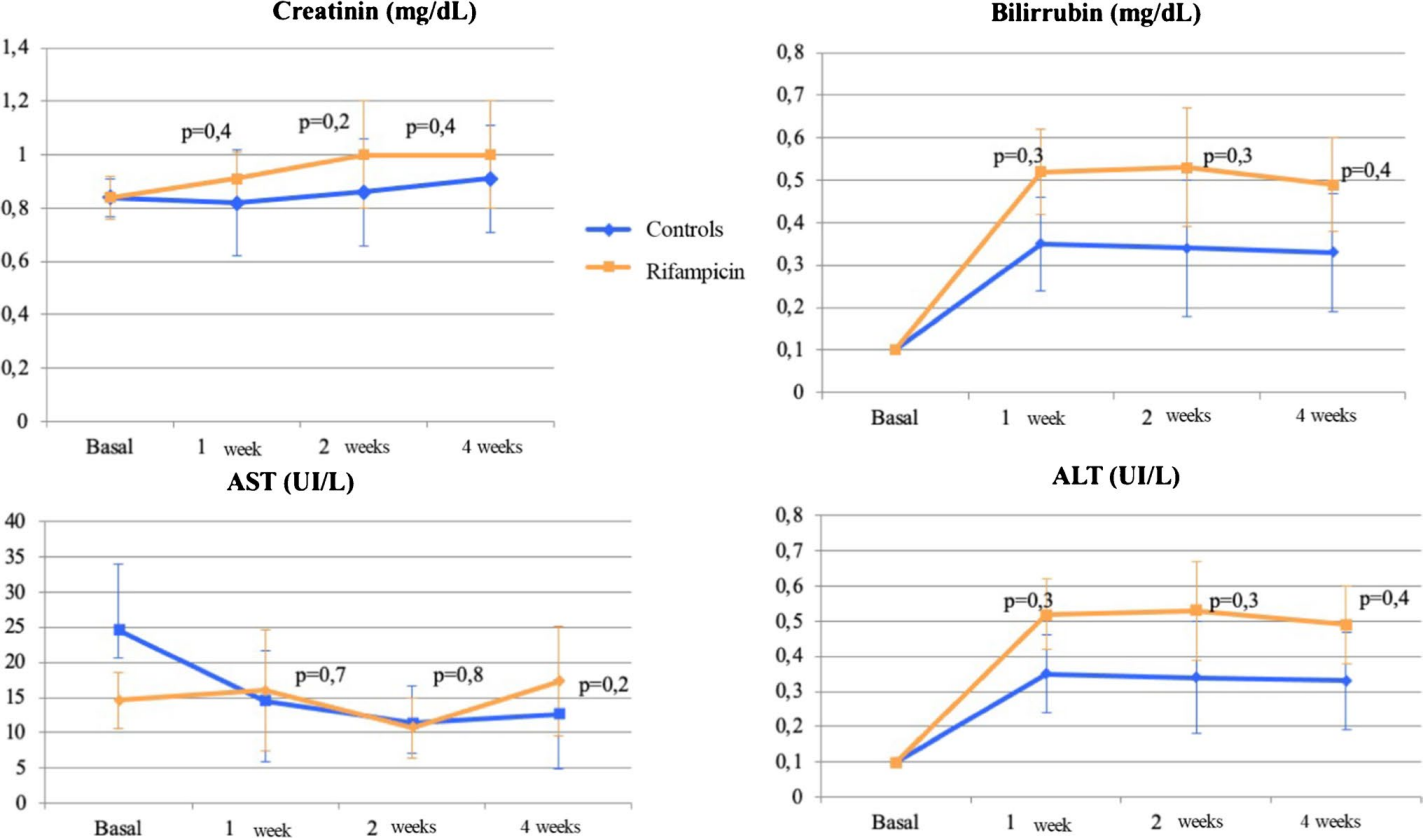
Evolution of toxicity parameters from baseline to the end of the study: **A** aspartate aminotransferase (AST), **B** alanine aminotransferase, **C** bilirubin, and **D** creatinine. Rifampicin group results are represented in orange and control group results in blue

Regarding the systemic toxicity of rifampicin, no statistically significant differences were found between the study groups in terms of AST, ALT, bilirubin, or creatinine values at any time during the study (Fig. 3). However, an elevation in bilirubin above normal values was detected in the R group, but this remitted spontaneously 2 weeks after placement of the spacer.

### Intra-articular release profile of microencapsulated rifampicin

**Fig. 4:**
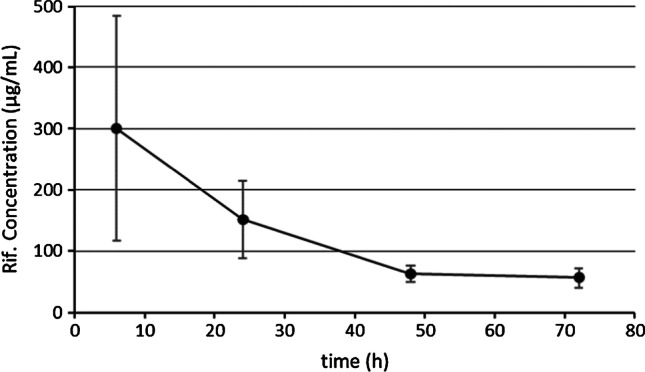
In vivo evolution of the intra-articular concentration of the rifampicin contained in microcapsules measured by HPLC

The analysis of intra-articular samples showed the release of rifampicin from the cement spacer at higher concentrations than the staphylococcal MIC throughout the study. The mean highest concentration registered was 594.79 μg/mL at 6 h postoperatively. In addition, the obtained release curve showed an initial rapid release phase followed by a slower ‘plateau’ phase (Fig. 4). Intra-articular sample collection could not be performed beyond 72 hours post-operatively given the absence of fluid in the animals’ knees, probably due to the decrease in inflammation associated with the infection.

## Discussion

Although PJI is an old acquaintance of the orthopaedic surgeon, the therapeutic strategies available are far from being considered highly effective for its eradication. The rate of synthesis of new antibiotics has been surpassed by the speed of appearance of resistant microorganisms, so a good treatment strategy is the revision of the efficacy spectra of known antimicrobials. The importance of systemic rifampicin in the treatment of staphylococcal PJI is widely documented, having demonstrated its action against bacteria in stationary phase, intracellular forms, and its diffusion capacity in biofilm, thus improving the results of PJI treatment when it is used in combination with other antibiotics [13, 22], but the systemic toxicity limits the doses used in clinical practice. These circumstances make research into new tools that allow the intra-articular release of rifampicin from bone cement particularly important.

In 2016, our research group designed a control release delivery system of rifampicin that keeps it isolated from the bone cement during the polymerisation phase, allowing complete setting of the bone cement in usual times of work. In addition, the incorporation of rifampicin into microcapsules allows its controlled release, which, predictably, would result in levels above the minimum effective concentration for a prolonged period of time [16–18]. In the present work, we carried out the in vivo assessment of the efficacy and safety of the rifampicin microcapsules.

To determine the efficacy of cement containing rifampicin microcapsules, a two-stage replacement was reproduced comparing cement containing gentamicin used in routine clinical practice with and without the addition of rifampicin microcapsules. The rifampicin was combined with another antibiotic to minimise the appearance of resistances, in which rapid development is the main problem associated with its use in monotherapy [7, 23]. In order to discriminate between the antimicrobial effect produced by the microencapsulated rifampicin from the produced by gentamicin the same cement with gentamicin was used in the control group. To avoid errors in the assessment of the efficacy and toxicity, no intravenous antibiotics were administered. The microbiological analysis revealed statistically significant differences between groups in terms of the number of CFUs isolated in bone and soft tissue, since all the samples from the group treated with rifampicin microcapsules showed no bacterial growth, whereas three animals from the control group showed positive cultures in the bone samples and four in the soft tissue samples. These results demonstrate the superiority of cement containing gentamicin and rifampicin microcapsules in the eradication of PJI.

Due to the classic impossibility of adding rifampicin to bone cement, there is no evidence on the systemic absorption of intra-articular rifampicin included in bone cement, so that no side effects associated with this administration route have been recorded either. Our study’s toxicity determination was carried out by monitoring the major side effects of rifampicin when used systemically: hepatotoxicity and flu-like syndrome.

Hepatotoxicity is a side effect which results in hepatic alanine aminotransferase (ALT) elevation. Although ALT values were higher in group R than in group C (Fig. 3), these differences were no statistically significant and did not exceed 80 IU/mL in any of the animals, which corresponds to a grade 0 hepatotoxicity [24]. An increase in bilirubin levels was found in the rifampicin group, exceeding the 0.5 mg/dL limit of normality and corresponding to a cholestasis pattern [25]. This increase was considered the development of an asymptomatic cholestatic jaundice, which normalised spontaneously in the second week of the study (Fig. 3). The cholestatic jaundice is a transient side effect with no clinical relevance due to competition between bilirubin and rifampicin for the hepatocyte receptor [26].

The flu-like syndrome is a hypersensitivity reaction characterised by the appearance of urticaria, thrombocytopenic purpura, leukopenia, haemolysis, and renal failure secondary to glomerulonephritis or interstitial nephritis [7]. The assessment of urticaria and purpura was not feasible given the characteristics of the animals: body covered by hair and frequent grooming. No thrombocytopenia, leukopenia, or anaemia were detected in any of the animals; in fact, there was an increase in the platelet and leukocyte count secondary to the infection, which steadily decreased without normalising after 4 weeks of spacer implantation in both groups without statistically significant differences between them. Renal failure was monitored by determination of plasma creatinine levels, without finding statistically significant differences between groups, with mean values remaining below 2.5 mg/dL throughout (Fig. 3).

As previously said, the biofilm reduces the activity of antibiotics, increasing the MIC and multiplying the active dose necessary for the eradication of the infection [6, 27]. Despite this, most studies aim to demonstrate the efficacy of an antibiotic contained in bone cement or in orthopaedic implants exclusively performing an in vitro calculation of the release kinetics without performing in vivo tests [28–30]. In the present study, we carried out an in vivo determination of the intra-articular release curve of rifampicin contained in microcapsules added to the bone cement. It is challenging to define the intra-articular release kinetics of rifampicin from bone cement because the amount of synovial fluid varies among animals and between moments at which the extraction is performed. We were, however, able to demonstrate that the release of rifampicin is maintained at least for 72 hours, with concentrations higher than the in vivo MIC for *S. aureus* (0.06 μg/mL) [31, 32]. This finding is especially relevant because the exposure to sub-inhibitory concentrations of antibiotic leads to resistance development by the microorganisms contained in the biofilm [33]. The release curve was analogous to those reported in the literature for other antibiotics, with an initial peak of faster release and progressive decrease until reaching a plateau [34–36]. In all the determinations, our release curve showed intra-articular rifampicin concentrations higher than those detected by Anguita-Alonso et al. in 2006 with the use of non-encapsulated rifampicin [37].

The main limitation of the present study is the small sample size, an increase of which would improve the validity of our results and enable identification of low-incidence side effects. Despite these, current regulations consider essential the reduction of the number of animals used in preclinical trials, so only three animals more than the minimum of 6 per group were used. The use of three more animals is justified as precaution against the risk of losing animals due to systemic complications of the infection, antibiotic toxicity, or complications during its handling.

In conclusion, our results reveal cement containing rifampicin microcapsules as an effective treatment strategy for PJI caused by *Staphylococcus aureus*, showing intra-articular concentrations above the staphylococcal MIC without systemic toxicity that contraindicate its use in clinical practice. Therefore, the incorporation of microencapsulated rifampicin into bone cement is considered a safe form of local administration of high concentrations of antibiotic with less toxicity than the systemic route. However, further studies are necessary in order to expand sample size and improve result extrapolation quality and to perform preclinical acute toxicity tests to define the maximum rifampicin dose for inclusion in the PMMA.

## Data Availability

The data that support the findings of this study are available from the corresponding author, IILT, upon request.
